# Simple System for Isothermal DNA Amplification Coupled to Lateral Flow Detection

**DOI:** 10.1371/journal.pone.0069355

**Published:** 2013-07-26

**Authors:** Kristina Roskos, Anna I. Hickerson, Hsiang-Wei Lu, Tanya M. Ferguson, Deepali N. Shinde, Yvonne Klaue, Angelika Niemz

**Affiliations:** 1 Keck Graduate Institute of Applied Life Sciences, Claremont, California, United States of America; 2 Claremont BioSolutions, Upland, California, United States of America; San Francisco General Hospital, University of California San Francisco, United States of America

## Abstract

Infectious disease diagnosis in point-of-care settings can be greatly improved through integrated, automated nucleic acid testing devices. We have developed an early prototype for a low-cost system which executes isothermal DNA amplification coupled to nucleic acid lateral flow (NALF) detection in a mesofluidic cartridge attached to a portable instrument. Fluid handling inside the cartridge is facilitated through one-way passive valves, flexible pouches, and electrolysis-driven pumps, which promotes a compact and inexpensive instrument design. The closed-system disposable prevents workspace amplicon contamination. The cartridge design is based on standard scalable manufacturing techniques such as injection molding. Nucleic acid amplification occurs in a two-layer pouch that enables efficient heat transfer. We have demonstrated as proof of principle the amplification and detection of *Mycobacterium tuberculosis* (*M.tb*) genomic DNA in the cartridge, using either Loop Mediated Amplification (LAMP) or the Exponential Amplification Reaction (EXPAR), both coupled to NALF detection. We envision that a refined version of this cartridge, including upstream sample preparation coupled to amplification and detection, will enable fully-automated sample-in to answer-out infectious disease diagnosis in primary care settings of low-resource countries with high disease burden.

## Introduction

Nucleic acid amplification testing (NAAT) can diagnose infectious diseases by identifying the pathogen's genetic material [1], [2]. NAAT is usually performed in centralized laboratories by highly-trained personnel on large, complex, expensive equipment, which is not ideal for applications that require a rapid answer to facilitate treatment and improve patient outcomes [3]–[5]. In developing countries, the diagnosis of endemic infectious diseases through NAAT in centralized laboratories is further hampered by lack of suitable facilities and trained personnel, and additional logistical challenges [6]–[8]. Our goal is to develop a fully-integrated NAAT device to diagnose infectious diseases at the point of care (POC) in low-resource settings. As a step towards this goal, we present proof of principle for an early prototype of a disposable, pouch-based cartridge to execute DNA amplification and detection, in conjunction with a compact, inexpensive, low-power heater.

The GeneXpert (Cepheid, Sunnyvale, CA) exemplifies the move of NAAT towards the point of care [9]. This fully-automated and integrated bench-top system can perform sample preparation, nucleic acid amplification via polymerase chain reaction (PCR), and multi-channel real time fluorescence detection in less than 2 hours [10]–[12]. However, the size, cost, and maintenance requirements of the GeneXpert instrument may impede broad-scale implementation in resource-limited high-burden countries [13]. Other PCR-based fully- or partially-integrated NAAT systems for infectious disease diagnosis are in development or on the market [14]–[17], but are relatively expensive systems due to the complexity associated with thermocycling and real-time fluorescence detection. Isothermal NAAT requires a single reaction temperature, and, therefore, utilizes simplified instrumentation compared to real-time PCR. In recent years, significant progress has been made in automating isothermal amplification methods in a miniaturized format [18]–[21]. Several isothermal NAAT assays and systems have entered the US market for infectious disease diagnosis in moderate complexity clinical laboratories [22]–[24], with varying levels of process integration, system complexity, and ease of use.

Isothermal amplification can be coupled with real time detection, as exemplified by the Meridian IllumiGene® system which employs LAMP as the method of nucleic acid amplification and real-time detection from clinical samples [22]. However, real time detection increases instrument cost and complexity. Several visual end point detection formats have been reported for LAMP, based on changes in turbidity [25] or fluorescence [26]. These approaches are reasonably simple to perform, do not require open handling of amplified master-mix, thereby minimizing the risk for amplicon carryover, and have been field tested for TB diagnosis in low-resource settings [26]. However, the readout can be ambiguous, especially at lower target concentrations and at varying levels of ambient light. Readout based on fluorescence requires a UV light source and additional manual tube handling after amplification, and does not lend itself to full process integration.

Nucleic Acid Lateral Flow (NALF) is emerging as an alternative simple and inexpensive endpoint detection method. Lateral flow devices are well established for POC diagnostics, can be manufactured inexpensively in large quantities, rely on passive fluidics, and provide a clear visual readout with no additional instruments required. NALF has been coupled to PCR [27] and to many isothermal amplification methods [28]–[33]. NAAT systems for infectious disease diagnosis commercialized by BioHelix [23] and USTAR [28] use a NALF cassette to analyze the amplified master-mix. This NALF cassette completely contains the amplified master-mix to avoid amplicon carry-over contamination, which is a major challenge in NAAT. However, in these systems, NALF detection is performed as a separate manual step, after isothermal amplification has been executed on a standard heat block.

To integrate isothermal DNA amplification with NALF detection in one device requires a system that can heat the master-mix at a fixed temperature and set time, and then pump the amplified master-mix onto the lateral flow strip. While isothermal heating can be readily accommodated in a compact inexpensive instrument, fluid handling on disposable chips or inside cartridges typically requires bulky and complex positive displacement pumping systems. For example, the Cepheid GeneXpert [10] uses a mechanically actuated piston with a rotating valve. Other systems including the CARD (Chemistry and Reagent Device, Rheonix, Ithaca, NY), the LIAT (lab in a tube, IQuum, Marlborough, MA), the Razor and Film Array systems (Idaho Technologies, Salt Lake City, Utah), and the Portrait analyzer (Great Basin Scientific, Salt Lake City, Utah) utilize pneumatic or mechanical actuation for pumping [16], [17], [24], [34], [35]. Alternatively, electrolysis provides an inexpensive mechanism to pump fluids by using hydrogen and oxygen gases generated by water electrolysis to exert pressure on a downstream fluid [36]. Electrolytic pumping has been used for automated NAAT inside microfluidic devices developed by Motorola [37] and Combimatrix [38], [39].

Our ultimate goal is to enable fully-integrated, sample-in to answer-out diagnosis at the point of care and in low-resource settings using a compact and low-cost device. As a first step towards this goal, we have developed an early prototype for a disposable, pouch-based amplification and detection cartridge, in conjunction with a compact, inexpensive instrument that contains low-power electronics. For this proof of principle study, we have implemented either isothermal Loop-Mediated Amplification (LAMP) or Exponential Amplification (EXPAR) reactions to detect *M.tb* genomic DNA, coupled with NALF visual endpoint detection in the prototype cartridge. In a parallel effort, we are developing a module for nucleic acid sample preparation. Future work will focus on coupling sample preparation with amplification and detection into a single cartridge.

## Experimental

### System Design and Operating Concept

**Figure 1:**
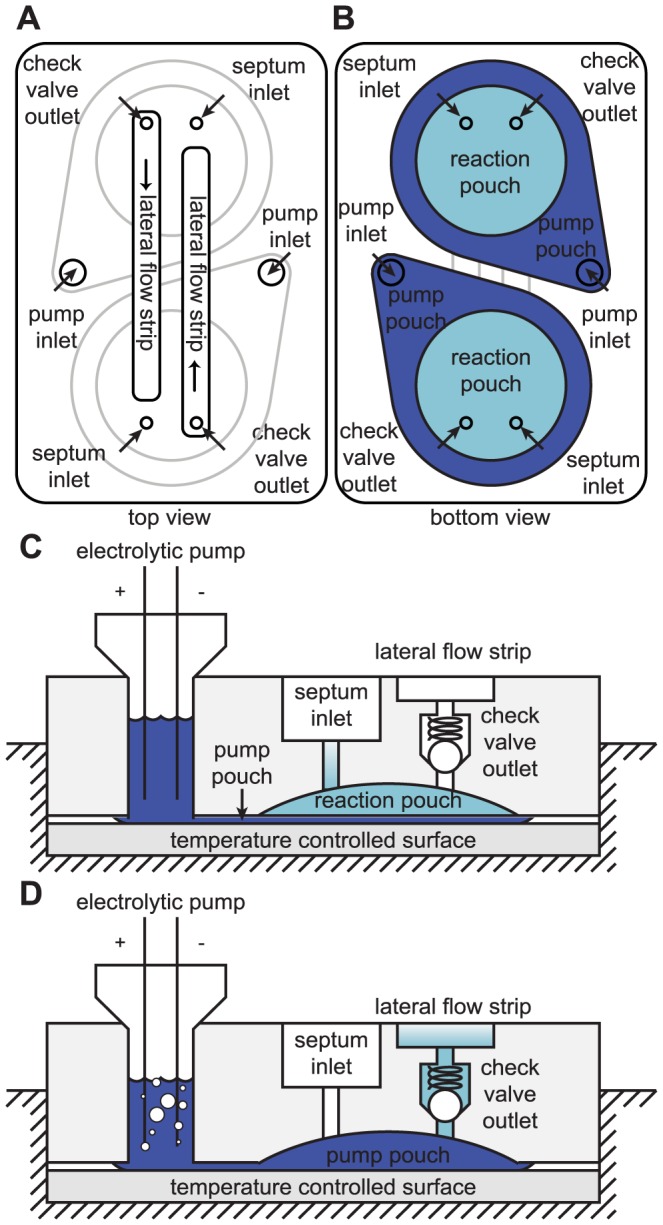
Cartridge concept. (a) Cartridge viewed from the top, showing access ports to the reaction and pump pouches on the underside of the cartridge (indicated in gray outline), and the position of lateral flow strips in anti-parallel orientation. (b) Cartridge viewed from the bottom, showing reaction pouches with overlaid pump pouches. (c) and (d) Side view of the cartridge on the heater (not to scale), illustrating the operating principle. (c) Master-mix is injected through the septum inlet into the reaction pouch, and targeted DNA sequences are isothermally amplified on top of the temperature-controlled heater surface. (d) Applying current to the electrolytic pump pushes fluid into the pump pouch. The pump pouch expands, thereby compressing the reaction pouch against the dome-shaped recess in the cartridge, forcing fluid out of the reaction pouch through the one-way check valve and into the lateral flow strip pouch.

Our system design, illustrated in Figure 1, allows for two samples to be tested simultaneously. The disposable cartridge contains on its top side (Figure 1a) two lateral flow strips in anti-parallel orientation, two septum inlets, and two attachment ports for electrolytic pumps. Two reaction pouches are attached to the bottom side of the cartridge (Figure 1b), enclosed by two slightly larger pump pouches which are sealed on top of the reaction pouches and connected to the electrolysis chamber ports. During process execution, an empty cartridge is attached to the handheld heating and electronics unit, where the cartridge is pre-heated to the reaction temperature. Reaction master-mix is injected from the top (Figure 1c) through the septum inlet port into the reaction pouch, which initiates isothermal DNA amplification. Once the reaction is completed, current is applied to the electrodes (Figure 1d) and the pump chamber is pressurized with gas produced via electrolysis, pumping the fluid within the reaction pouch through the outlet port and onto the lateral flow strip. The reaction mixture migrates along the strip based on passive capillary action, producing a visual readout. All fluids remain sealed within the cartridge, eliminating amplicon carry-over contamination.

### Cartridge Fabrication

The cartridge uses low-cost components and manufacturing techniques to meet the cost constraints of POC testing performed in low-resource settings. The main cartridge components are manufactured using injection molding and thermal bonding, both inexpensive and scalable techniques.

**Figure 2:**
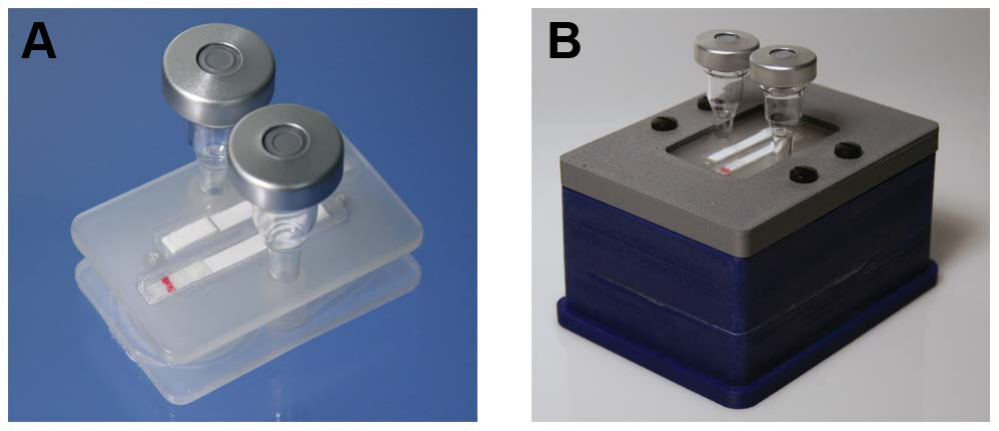
System components. (a) Top side of cartridge: lateral flow strips are sealed inside the grooves forming the lateral flow strip pouches, and electrolytic chambers are press-fit in place. (b) Heater with cartridge secured in place, prior to test execution. (a) and (b) The red line on the NALF strips represents the colored microspheres immobilized on the treated conjugate pad.

The cartridge consists of two polypropylene cards, with hollow protrusions in matching locations, which can be joined to form fluid conduits. One-way passive ball-and-spring valves (Lee Company, Westbrook, CT) were pressed into the hollow protrusions of the bottom card (Figure 1a, check valve outlet). Small cylindrical silicone septa were press-fit into the fluid conduits between the top and bottom cards, providing a leak-proof seal on the inlet through which master-mix can be injected into the reaction pouch using a needle and syringe (Figure 1a, septum inlet). Flexible polypropylene film was heat-sealed onto the bottom card to create two reaction pouches overlaid by two pump pouches. Lateral flow strips were inserted into grooves on the top card, and polypropylene film was heat-sealed onto the card to create two lateral flow strip pouches (Figure 2a). After attaching the pouches, the two cards were pressed together, creating snap-fit seals and leak-proof fluid conduits between the two cards. We inserted silicone foam between the two cards for insulation. To create the electrolysis chambers on the top of the cartridge, two large reservoirs were press fit into the fluid conduits that provide access to the pump pouch (Figure 1a, pump inlet, Figure 2a). We used stainless steel syringe needles to fill the electrolysis chambers and pump pouches with electrolyte solution. The needles were then capped closed, but were left in the electrolysis chambers to be used as electrodes. The needles used in the electrolytic pumps, and for injecting the sample through the inlet septum, were included as an intermediate solution in this preliminary cartridge iteration. In future refined cartridge designs, the needles in the pump chambers will be replaced with insert-molded electrodes, and a luer-type inlet port will be used for sample introduction. The cost of all cartridge components, excluding master-mix reagent materials, totals $5.20 per cartridge, of which $3.20 originates from the two check valves, and $1.20 originates from the currently used electrolysis chamber setup. In future design iterations [40], we are replacing these check valves with a far less expensive and more compact custom valve design, and are integrating the electrolysis chambers into the injection-molded cartridge body, which will significantly reduce the cartridge cost. Furthermore, this cost estimate refers to low-volume production, and will decrease further upon scale-up. The assembled cartridge measures approximately 2.3″ long, 1.55″ wide and 2″ deep, including the height of the electrolysis chambers (0.6″ without the electrolysis chambers).

### Portable Heater Fabrication

We designed and fabricated a stand-alone compact heater (approximately 4″ wide, 3″ long and 1.5 inches deep) to enable isothermal DNA amplification within the reaction pouches on the bottom side of the cartridge (Figure 2b). This system does not require an external computer for process execution. The temperature set point can be adjusted to accommodate different isothermal amplification reactions, e.g. 63°C for LAMP and 55°C for EXPAR. The chassis of the heater instrument was printed using a fused deposition modeling machine. Thin-film polyimide heaters were attached to the underside of an aluminum plate that acts as the heater surface within the device, to provide evenly-distributed heating. Polyurethane foam sealant inside the heater minimizes the power required to reach and maintain the reaction temperature. We controlled the temperature with a custom electronic circuit on a printed circuit board (PCB) using feedback from a thermistor embedded in the aluminum plate. The instrument used ∼8 W during the initial ∼3 minutes heating ramp, and ∼0.5 W to maintain the temperature. The low-power electronics in this system can be readily adapted to be operated using a rechargeable battery, eliminating the dependence on line power and increasing the potential for adoption of this system in rural health clinics.

### Isothermal Amplification Reactions

As target for the isothermal amplification reactions, we used *M.tb* genomic DNA (H37Ra, ATCC 25177D). LAMP reactions targeting the *M.tb* gyrB gene were performed using a modified version of the protocol reported by Iwamoto et al. [41]. To facilitate the detection of LAMP amplicons via lateral flow, the backward inner primer (BIP) was 5′-labeled with digoxigenin, and the forward loop primer (FLP) was biotinylated at the 5′ end. In separate experiments, we determined that LAMP assay performance is not adversely affected by using labeled primers (data not shown). As an alternative isothermal amplification method, we designed an EXPAR assay to detect a 38 bp sequence within the *M.tb* genome. Based on *in silico* analysis, this sequence is conserved within all *M.tb* genomic sequences deposited in NCBI, but not cross-reactive with non-tuberculous mycobacteria and other pathogens that may give rise to similar clinical symptoms as *M.tb*. We performed fingerprinting and two stage EXPAR using a modification of the reaction scheme previously described [42]. In the assay reported herein, we implemented NALF for the detection of reporter oligonucleotide Y generated by two-stage EXPAR in lieu of nanosphere aggregation. Oligonucleotide sequences utilized in these LAMP (Supporting Information Table S1) and EXPAR assays (Supporting Information Table S2) coupled to NALF, and other general reaction conditions (Supporting Information Text S1) for isothermal DNA amplification in the cartridge, are described in the supporting information.

### NALF Test Strip Fabrication

NALF test strips were fabricated in house using synthetic probe and capture oligonucleotides. For LAMP-NALF, oligo d(T)_90_ and neutravidin were conjugated respectively to red-dyed carboxylate modified polystyrene microspheres. For EXPAR- NALF, a probe complementary to the 3′ end of the EXPAR reporter Y oligonucleotide created in the reaction was conjugated to the red microspheres in a similar fashion. The microspheres were then dispensed and dried onto the NALF conjugate pad, which was pre-treated with a proprietary conjugate-releasing solution, using a lateral flow reagent dispenser (Claremont BioSolutions, Upland, CA).

For detection of LAMP amplicon, an anti-Digoxin antibody and a biotinylated d(A)_30_ oligonucleotide coupled to neutravidin were dispensed onto a nitrocellulose membrane as test and control lines, respectively. For EXPAR amplicon detection, biotinylated test and control oligonucleotides coupled to neutravidin were dispensed onto nitrocellulose, with the test line probe complementary to the 5′ end of the EXPAR reporter Y oligonucleotide, and the control line probe complementary to the probe conjugated to the microspheres.

To prepare test strips, the dispensed nitrocellulose membrane was mounted onto an adhesive plastic backing. The treated glass fiber conjugate pad and a cellulose fiber absorbent pad were attached to the bottom (sample) and the top portions of the adhesive backing respectively, each with a 1–2 mm overlap with the nitrocellulose membrane. The conjugate pad also extended 1–2 mm over the bottom edge of the adhesive backing to allow for proper fluidic contact within the cartridge. The resulting sheet was cut into 4 mm×50 mm individual test strips for assembly into the cartridge.

## Results and Discussion

### Fluidic Control

This cartridge utilizes electrolytic pumping and, therefore, does not require external pistons or actuators, as opposed to other clinical diagnostic systems using flexible pouches [16], [17], [24]. Similar to previous reports [36], we have observed a roughly linear relationship between applied current and flow rate for electrolytic pumping. To ensure that the lateral flow strip performs properly, we determined that amplified master-mix needs to be pumped from the reaction chamber into the lateral flow strip chamber at a flow rate of approximately 100 µL/min, which was obtained by applying 50 mA of current.

One-way valves are necessary to prevent the fluid within the reaction chamber from leaking prematurely into the lateral flow detection chamber. Injecting 75–100 µL liquid into the reaction chamber results in <2 psi fluid pressure, which is less than the cracking pressure of the valves (∼2 psi) used in the current design. Therefore, fluid is retained in the reaction pouch until electrolytic pumping forces the fluid through the passive valve and onto the lateral flow strip.

### Instrument Design and Thermal Control

**Figure 3:**
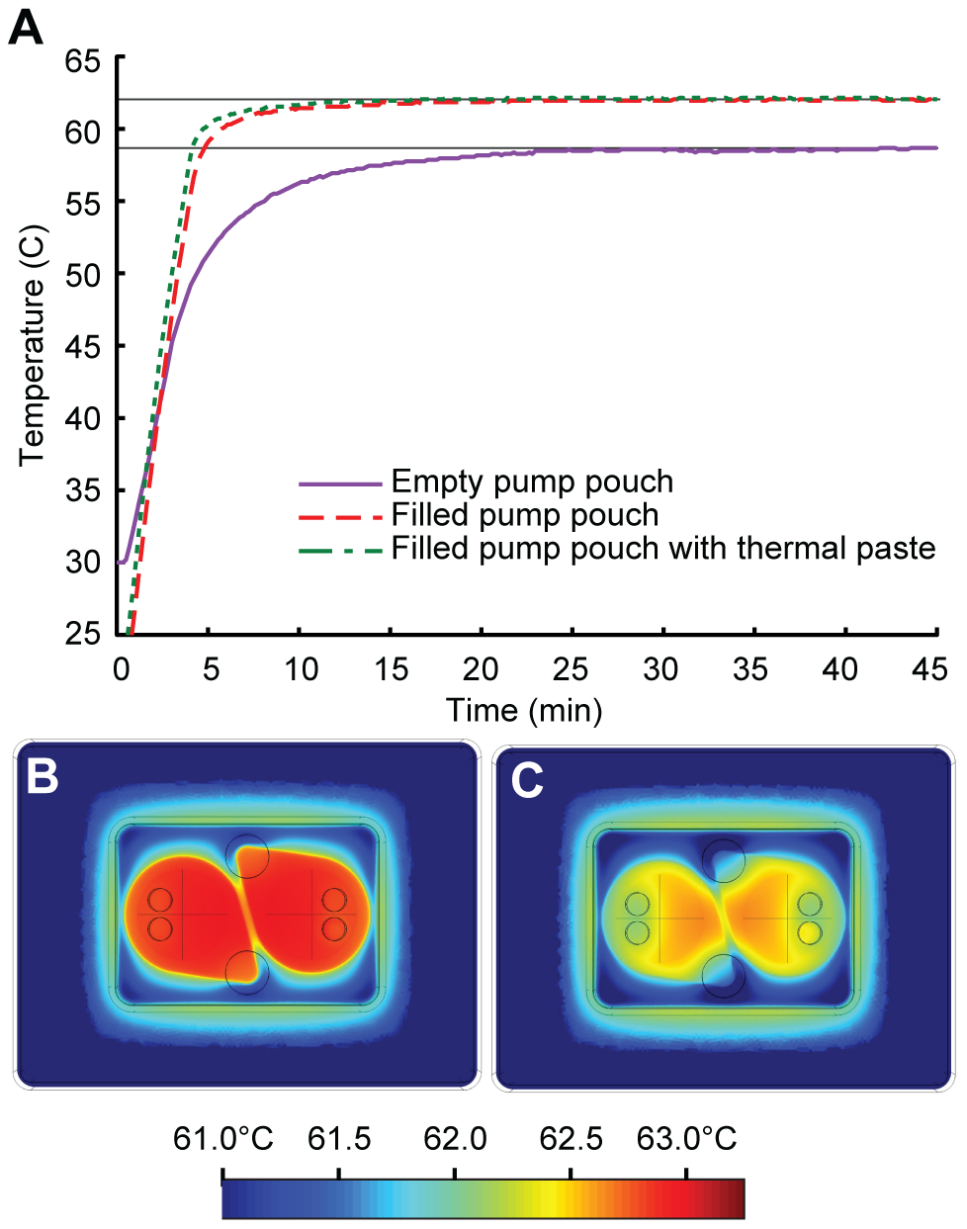
Thermal control within the cartridge. (a) Measured temperature of the fluid inside the reaction pouch of a cartridge attached to the heater as a function of time. Filling the pump pouch with liquid improves the thermal transfer from the heater to the reaction fluid, which reaches a stable temperature of 62±0.1°C within approximately ten minutes. Thermal paste applied between the heater surface and pump pouch provides no further improvements. (b) and (c) Thermal simulations (Comsol Multiphysics) of the cartridge on top of the heater, showing horizontal cross-sections within the center of the reaction fluid layer of the cartridge. (b) Uniform heating to the desired temperature (63°C) is observed using a model with ideal thermal contact between cartridge and heater, with no air gap. (c) Introducing a 150 µm thick thermally-resistive layer between cartridge and heater leads to a lower temperature and less uniform heating within the reaction pouch. The cross-hatches in the middle of the pump pouches indicate the dimensions of the circular reaction pouches. An air bubble was intentionally introduced into the outlet port of the reaction chamber to simulate trapped air in the reaction pouches after fluid is inserted.

Maintaining an appropriate and uniform temperature throughout the reaction pouch during the amplification period is essential for assay performance. With a cartridge inserted into the device, the heater surface requires approximately three minutes to heat from room temperature to 63°C and maintains this constant temperature to within ±0.1°C for as long as the heater is turned on. Comparable temperature stability is obtained at other temperatures, e.g. 55°C required for EXPAR. Air trapped between the fluid inside the reaction pouch and the heater significantly lowers the thermal conductivity across this interface, and can contribute to non-uniform fluid heating within the reaction pouch. This concern can be mitigated by filling the pump pouch with fluid. The slightly inflated pump pouch presses up against the heater surface, which compensates for imperfections on the cartridge bottom surface that cause the cartridge to not sit completely flat on the heater. Additionally, the fluid in the pump pouch effectively conducts heat to the reaction pouch. We measured the temperature of liquid in the reaction pouch using a thermocouple inserted through the inlet septum. If the pump pouch is empty, then the fluid temperature in the reaction pouch deviates significantly from the temperature measured directly on the heater surface underneath the cartridge (Figure 3a). Filling the pump pouch improves the thermal transfer efficiency between heater and cartridge, resulting in temperature equilibration after ∼10 minutes, and a final temperature closer to the desired set point. Thermal paste applied between the heater surface and pump pouch provides no further improvements. In practice, a cartridge with empty reaction pouches but filled pump pouches is pre-heated on the heater unit for at least ten minutes to allow the cartridge and instrument to thermally equilibrate. The master-mix is then injected into the reaction pouches, where it quickly reaches the desired reaction temperature. Therefore, the ten minute ramp up time does not adversely affect the amplification kinetics. In the envisioned fully integrated cartridge, this ten minute temperature ramp up will occur while sample preparation is in process in the upstream regions of the cartridge and therefore does not compromise overall process time.

We performed numerical simulations of the three-dimensional temperature profile as a function of three factors: the overall system design, the choice of materials, and the thermal contact between cartridge and heater. The model treats the thin-film heater as a two-dimensional heat source with a specified output power. The heat conducts through the interior of the cartridge and dissipates to the room temperature environment by convection to still air through the top surface of the cartridge, or through insulating material on the sides of the cartridge. Convection through still air is imposed using a natural convection boundary with a Dirichlet heat flux based on: N_V_ = (5 W)/(m^2^ K).

Using this model, we found that the temperature within the reaction pouches deviated from 63°C by less than 0.5°C throughout the reaction chamber if the cartridge makes ideal contact with the heater surface (Figure 3b). To understand the effects of non-ideal thermal contact, we modeled the system with a 150 µm thick thermally resistive air layer between the pump pouches and the heater surface (Figure 3c). This resistive layer significantly reduced the heat transfer between the heater and cartridge, decreasing the maximum temperature in the reaction pouches by 1°C, and leading to more significant temperature variation throughout the reaction pouch.

### Isothermal Amplification Coupled to NALF Detection in the Cartridge

**Figure 4:**
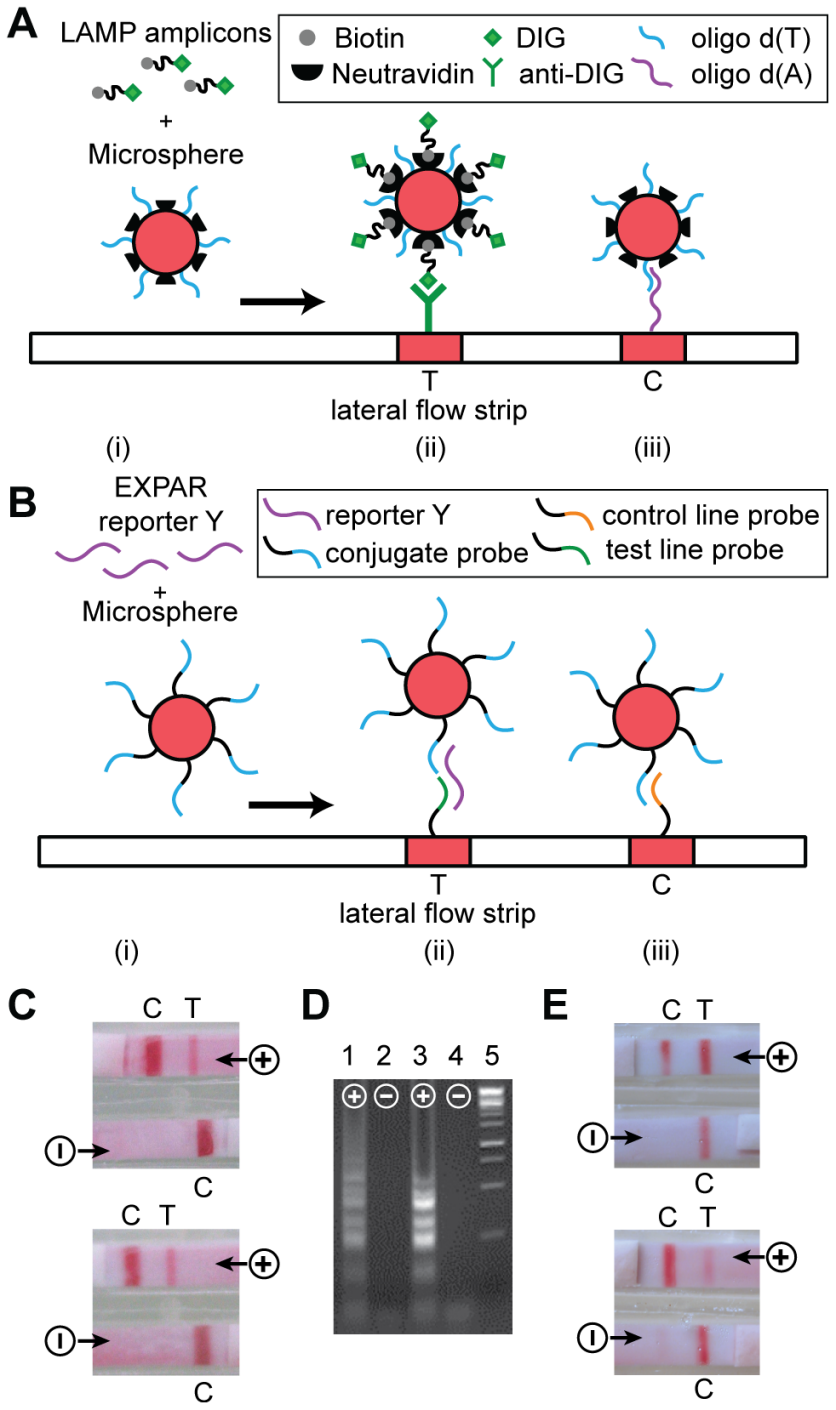
Isothermal DNA amplification coupled to NALF detection. Conceptual depiction for (a) LAMP, and (b) EXPAR. (i) amplified master-mix applied to the conjugate pad enables amplicons to interact with colored polystyrene microspheres functionalized with appropriate capture moieties. (ii) After migrating along the nitrocellulose membrane, microspheres carrying amplicons are captured at the test line. (iii) At the control line, microspheres are captured irrespective of the presence of amplicon. (c) LAMP based detection of *M.tb* genomic DNA performed in the cartridge on the instrument: NALF strips of two representative cartridges, after 10 minutes of isothermal amplification, followed by 10 min for lateral flow detection. (d) LAMP master-mix amplified in the cartridge on the heater, analyzed via gel electrophoresis (Lanes 1 and 2), compared to amplification performed in reaction tubes on a standard heat block (Lanes 3 and 4). Lane 5: DNA molecular weight markers. For (c) and (d), positive (+) reactions show LAMP product starting from 3000 copies of *M.tb* DNA, and negative (−) reactions show no product since no *M.tb* DNA was added to the reaction. (e) EXPAR based detection of *M.tb* genomic DNA performed in the cartridge on the instrument: NALF strips of two representative cartridges, after 60 minutes of isothermal amplification, followed by 10 min for lateral flow detection. Positive (+) reactions contained 6×10^5^ copies *M.tb* DNA, and negative (−) reactions contained no *M.tb* DNA.

To establish proof of principle that isothermal amplification coupled to lateral flow detection can be executed in the cartridge on the heater device, we chose two different isothermal amplification reactions that detect *M.tb* genomic DNA as the target. The first reaction is an established, clinically-validated LAMP assay that has been used for TB diagnosis in low resource settings [26], [41]. We have modified this assay to enable coupling with NALF detection (Figure 4a). Coupling LAMP to NALF has been demonstrated for other assays [32], but the complicated, large amplicon structure can cause steric hindrance that may result in compromised line intensities and test results. Our approach for LAMP amplicon detection via NALF uses a somewhat different reaction scheme. In positive reactions, the LAMP master-mix contained biotin/DIG-labeled amplicons. Through the biotin moiety, amplicons were captured by the neutravidin-conjugated microspheres. After migrating along the nitrocellulose strip, microspheres carrying LAMP amplicons were captured at the test line (T), based on the interaction between the DIG-labeled portion of the LAMP product and the immobilized anti-DIG antibody (Figure 4a(ii)). At the control line (C), the oligo-d(A) immobilized on the nitrocellulose membrane hybridized to the oligo-d(T) conjugated to the microspheres (Figure 4a(iii)). This control line confirms proper performance of the lateral flow strip, and should appear in positive and negative reactions as hybridization is independent of the presence of LAMP amplicons. In contrast, the test line should only appear in positive reactions. In addition, many NALF reactions require an extra liquid running buffer, which introduces another reagent and complicates the device design. In our LAMP-NALF scheme, amplified master-mix can be applied directly to the conjugate pad of the NALF strip, where it reconstituted a dry reagent mixture containing colored microspheres. No other liquid reagents are required other than the amplified master-mix.

The second assay used herein is based on the Exponential Amplification Reaction (EXPAR), which amplifies short trigger oligonucleotides at 55°C using polymerase and nicking enzyme activities [43]. In prior work, we have demonstrated that the trigger oligonucleotide amplified in EXPAR can be generated from a targeted genomic DNA sequence that contains adjacent nicking enzyme recognition sites through the Fingerprinting reaction [42], and have implemented EXPAR in a two stage reaction format to generate a reporter oligonucleotide that can be detected via a nanosphere based colorimetric readout [44]. We report herein the detection of *M.tb* genomic DNA through a Fingerprinting two-stage EXPAR assay with NALF-based readout. Two-stage EXPAR generates large amounts of single-stranded reporter oligonucleotide Y that can form a sandwich complex with complementary probe oligonucleotides conjugated to microspheres and to the test line of a NALF strip (Figure 4b). Therefore, the test line becomes visible in positive reactions. The probe oligonucleotide immobilized at the NALF control line hybridizes directly to the probe sequence conjugated to the microspheres. Therefore, the control line appears in positive and negative reactions. Again, we developed an EXPAR NALF strip with a dry reagent mixture in the conjugate pad, to which amplified master-mix can be applied directly without the need for additional liquid reagents.

We tested the performance of both isothermal assays coupled to NALF detection in the cartridge prototype. For these experiments, the heater set-point was adjusted for the desired reaction temperature (63°C for LAMP and 55°C for EXPAR), and a cartridge with empty reaction chambers was allowed to pre-heat on the heater for ten minutes. We then injected 100 µL master-mix through the inlet septum into the reaction chambers, with one positive and one negative reaction per cartridge. We allowed the reaction to incubate on the heater for 10 minutes (LAMP) or 60 minutes (EXPAR), then applied current to the electrolytic pumps, and allowed 10 minutes for NALF detection. The final readouts for LAMP-NALF (Figure 4c) and EXPAR-NALF (Figure 4e) consisted of the expected two lines for the positive and a single control line for the negative reactions. For LAMP, we also determined that master-mix amplified in the cartridge produces the same characteristic pattern of high molecular weight amplicons as control reactions performed under standard conditions (Figure 4d).

For LAMP, positive reactions contained 3000 copies of *M.tb* genomic DNA, while for EXPAR, positive reactions contained 6×10^5^ copies of *M.tb* genomic DNA. Relatively high concentrations of target genomic DNA were chosen as a means of establishing proof of principle for the cartridge prototype. The goal of the study reported herein was not to test the limit of detection for the assays. However, during assay development using a standard thermocycler for isothermal amplification in tubes we have successfully detected 3 copies of *M.tb* genomic DNA using LAMP coupled to NALF and 2×10^4^ copies of *M.tb* genomic DNA using EXPAR coupled to NALF, which was achievable after 70 minutes of amplification (data not shown). In all cases, negative reactions contained no *M.tb* genomic DNA, but all reactions included 10 ng human genomic DNA, which is present as background in clinical samples. The assays are therefore not cross-reactive with human genomic DNA. In the cartridge, LAMP produced results after 10 minutes of isothermal amplification, likely due to the relatively high starting copy number [41]. The EXPAR assay, which still needs to be optimized, required longer amplification under the current reaction conditions.

## Conclusions

Using the cartridge and portable, low-power heater reported herein, we have demonstrated detection of purified *M.tb* genomic DNA through isothermal LAMP and EXPAR reactions coupled with NALF in a fully-enclosed combined format. Through this early prototype, we obtained suitable thermal control, and have established proof of principle for the novel fluid handling approach using two-layer pouches combined with one-way valves and electrolytic pumping. This system is capable of executing different isothermal amplification reactions coupled with NALF detection, such as the LAMP and EXPAR assays described herein.

We are refining the design of this amplification and detection subunit [40] to incorporate miniaturized and integrated electrolytic pumps and novel passive valves, and to enable automated pumping, heating, and timing. For eventual use in point-of-care settings, it is imperative to couple extraction and purification of nucleic acids from clinical specimens with the herein described system for downstream isothermal DNA amplification and NALF detection. Therefore, in a parallel effort, we are developing a sample preparation subunit based on similar design principles, which performs mechanical lysis of mycobacteria [45] and DNA extraction from liquefied and disinfected sputum. Our next step is to combine sample preparation, amplification, and detection in a final integrated cartridge and device, to enable fully automated sample-in to answer-out diagnosis of active tuberculosis. At the current time, we estimate that the cost of all components for this integrated cartridge at moderate production volumes will be ∼$6 including master-mix reagents, lateral flow strips, and other reagents stored on-board the cartridge. We anticipate that these costs will be reduced further through component and manufacturing refinements, and upon scale-up. Similar to the preliminary system described herein, the refined system will not require an external computer to control process execution. The portable electronics unit for this refined system again is designed using low-power components to ensure eventual battery-powered operation in the field. We anticipate the refined cartridge to increase in size to approximately 2.25″ wide, 4.5″ long and 0.75″ deep, while the refined instrument will be approximately 5″ wide, 8″ long and 4″ deep.

Furthermore, this system has been designed as a platform technology that can be easily modified to accommodate different sample matrices during sample preparation, and different isothermal DNA amplification assays coupled to NALF detection. Such a universal platform technology is intended to enable the diagnosis of different pathogens in a fully-integrated and user-friendly format requiring minimal infrastructure, and is therefore suitable for use in near-patient low-resource settings.

## Supporting Information

Table S1 **Oligonucleotide Sequences for LAMP Amplification and NALF Detection.**
(DOCX)

Table S2 **Oligonucleotide Sequences for EXPAR Amplification and NALF Detection.**
(DOCX)

Text S1 **General Reaction Conditions for Isothermal Amplification Coupled to NALF Detection in the Cartridge.**
(DOCX)

## References

[1] Kaltenboeck B, Wang CM (2005) Advances in real-time PCR: Application to clinical laboratory diagnostics. Adv Clin Chem 40: 219–259 DOI10.1016S0065242305400062/S0065-2423(05)40006-2 16355924 10.1016/S0065-2423(05)40006-2 PMC7112074

[2] Ince J, McNally A (2009) Development of rapid, automated diagnostics for infectious disease: Advances and challenges. Expert Review of Medical Devices 6: 641–651 DOI10.1586ERD.09.46/ERD.09.46 19911875 10.1586/erd.09.46

[3] de Tejada BM, Stan CM, Boulvain M, Renzi G, Francois P, et al (2010) Development of a rapid PCR assay for screening of maternal colonization by group B Streptococcus and neonatal invasive Escherichia coli during labor. Gynecol Obstet Invest 70: 250–255 DOI10.1159000314014/000314014 21051844 10.1159/000314014

[4] Wolk DM, Marx JL, Dominguez L, Driscoll D, Schifman RB (2009) Comparison of MRSASelect agar, CHROMagar Methicillin-Resistant Staphylococcus aureus (MRSA) medium, and Xpert MRSA PCR for detection of MRSA in nares: Diagnostic accuracy for surveillance samples with various bacterial densities. J Clin Microbiol 47: 3933–3936 DOI10.1128JCM.0060109/JCM.00601-09 19828738 10.1128/JCM.00601-09 PMC2786641

[5] Brenwald NP, Baker N, Oppenheim B (2010) Feasibility study of a real-time PCR test for methicillin-resistant Staphylococcus aureus in a point of care setting. J Hosp Infect 74: 245–249 DOI10.1016j.jhin.2009.09.007/j.jhin.2009.09.007 19914735 10.1016/j.jhin.2009.09.007

[6] Yager P, Domingo GJ, Gerdes J (2008) Point-of-care diagnostics for global health. Annual Review of Biomedical Engineering 10: 107–144 DOI10.1146annurev.bioeng.10.061807.160524/annurev.bioeng.10.061807.160524 10.1146/annurev.bioeng.10.061807.160524 18358075

[7] Puren A, Gerlach JL, Weigl BH, Kelso DM, Domingo GJ (2010) Laboratory operations, specimen processing, and handling for viral load testing and surveillance. J Infect Dis 201: S27–S36 DOI10.1086650390/650390 20225943 10.1086/650390

[8] Stevens WS, Marshall TM (2010) Challenges in implementing HIV load testing in South Africa. J Infect Dis 201: S78–S84 DOI10.1086650383/650383 20225952 10.1086/650383

[9] Boehme CC, Nabeta P, Hillemann D, Nicol MP, Shenai S, et al (2010) Rapid molecular detection of tuberculosis and rifampin resistance. N Engl J Med 363: 1005–1015 DOI10.1056NEJMoa0907847/NEJMoa0907847 20825313 10.1056/NEJMoa0907847 PMC2947799

[10] Raja S, Ching J, Xi LQ, Hughes SJ, Chang R, et al (2005) Technology for automated, rapid, and quantitative PCR or reverse transcription-PCR clinical testing. Clin Chem 51: 882–890 DOI10.1373clinchem.2004.046474/clinchem.2004.046474 15746302 10.1373/clinchem.2004.046474

[11] Blakemore R, Story E, Helb D, Kop J, Banada P, et al (2010) Evaluation of the analytical performance of the Xpert MTB/RIF assay. J Clin Microbiol 48: 2495–2501 DOI10.1128JCM.0012810/JCM.00128-10 20504986 10.1128/JCM.00128-10 PMC2897495

[12] Helb D, Jones M, Story E, Boehme C, Wallace E, et al (2010) Rapid detection of Mycobacterium tuberculosis and rifampin resistance by use of on-demand, near-patient technology. J Clin Microbiol 48: 229–237 DOI10.1128JCM.0146309/JCM.01463-09 19864480 10.1128/JCM.01463-09 PMC2812290

[13] Mwaba P, McNerney R, Grobusch MP, O'Grady J, Bates M, et al (2011) Achieving STOP TB Partnership goals: Perspectives on development of new diagnostics, drugs and vaccines for tuberculosis. Tropical Medicine & International Health 16: 819–827 DOI10.1111j.13653156.2011.02777.x/j.1365-3156.2011.02777.x 21489070 10.1111/j.1365-3156.2011.02777.x

[14] Niemz A, Boyle DS (2012) Nucleic acid testing for tuberculosis at the point-of-care in high-burden countries. Expert Review of Molecular Diagnostics 687–701 DOI10.1586erm.12.71/erm.12.71 23153237 10.1586/erm.12.71 PMC3740172

[15] Niemz A, Ferguson TM, Boyle DS (2011) Point-of-care nucleic acid testing for infectious diseases. Trends Biotechnol 29: 240–250 DOI10.1016j.tibtech.2011.01.007/j.tibtech.2011.01.007 21377748 10.1016/j.tibtech.2011.01.007 PMC3746968

[16] Tanriverdi S, Chen LJ, Chen SQ (2010) A rapid and automated sample-to-result HIV load test for near-patient application. J Infect Dis 201: S52–S58 DOI10.1086650387/650387 20225947 10.1086/650387

[17] Poritz MA, Blaschke AJ, Byington CL, Meyers L, Nilsson K, et al (2011) FilmArray, an automated nested multiplex PCR system for multi-pathogen detection: Development and application to respiratory tract infection. Plos One 6 DOI10.1371journal.pone.0026047/journal.pone.0026047 10.1371/journal.pone.0026047 PMC3198457 22039434

[18] Asiello PJ, Baeumner AJ (2011) Miniaturized isothermal nucleic acid amplification, a review. Lab on A Chip 11: 1420–1430 DOI10.1039c0lc00666a/c0lc00666a 21387067 10.1039/c0lc00666a

[19] Mahalanabis M, Do J, ALMuayad H, Zhang JY, Klapperich CM (2010) An integrated disposable device for DNA extraction and helicase dependent amplification. Biomedical Microdevices 12: 353–359 DOI10.1007s1054400993918/s10544-009-9391-8 20066496 10.1007/s10544-009-9391-8 PMC2998058

[20] Wang CH, Lien KY, Wu JJ, Lee GB (2011) A magnetic bead-based assay for the rapid detection of methicillin-resistant Staphylococcus aureus by using a microfluidic system with integrated loop-mediated isothermal amplification. Lab on A Chip 11: 1521–1531 DOI10.1039c0lc00430h/c0lc00430h 21399774 10.1039/c0lc00430h

[21] Wu Q, Jin W, Zhou C, Han S, Yang W, et al (2011) Integrated glass microdevice for nucleic acid purification, loop-mediated isothermal amplification, and online detection. Anal Chem 83: 3336–3342 DOI10.1021ac103129e/ac103129e 21456520 10.1021/ac103129e

[22] Boyanton BL, Sural P, Loomis CR, Pesta C, Gonzalez-Krellwitz L, et al (2012) Loop-mediated isothermal amplification compared to real-time PCR and enzyme immunoassay for toxigenic Clostridium difficile detection. J Clin Microbiol 50: 640–645 DOI10.1128JCM.0101411/JCM.01014-11 22189114 10.1128/JCM.01014-11 PMC3295153

[23] Kim HJ, Tong YH, Tang W, Quimson L, Cope VA, et al (2011) A rapid and simple isothermal nucleic acid amplification test for detection of herpes simplex virus types 1 and 2. J Clin Virol 50: 26–30 DOI10.1016j.jcv.2010.09.006/j.jcv.2010.09.006 20947417 10.1016/j.jcv.2010.09.006 PMC3018672

[24] Hicke B, Pasko C, Groves B, Ager E, Corpuz M, et al (2012) Automated detection of toxigenic Clostridium difficile in clinical samples: Isothermal tcdB amplification coupled to array-based detection. J Clin Microbiol 50: 2681–2687 DOI10.1128JCM.0062112/JCM.00621-12 22675134 10.1128/JCM.00621-12 PMC3421499

[25] Mori Y, Notomi T (2009) Loop-Mediated Isothermal Amplification (LAMP): a rapid, accurate, and cost-effective diagnostic method for infectious diseases. Journal of Infection and Chemotherapy 15: 62–69 DOI10.1007s1015600906699/s10156-009-0669-9 19396514 10.1007/s10156-009-0669-9 PMC7087713

[26] Boehme CC, Nabeta P, Henostroza G, Raqib R, Rahim Z, et al (2007) Operational feasibility of using loop-mediated isothermal amplification for diagnosis of pulmonary tuberculosis in microscopy centers of developing countries. J Clin Microbiol 45: 1936–1940 DOI10.1128JCM.0235206/JCM.02352-06 17392443 10.1128/JCM.02352-06 PMC1933042

[27] Dineva MA, Candotti D, Fletcher-Brown F, Allain JP, Lee H (2005) Simultaneous visual detection of multiple viral amplicons by dipstick assay. J Clin Microbiol 43: 4015–4021 DOI10.1128JCM.43.8.4015.4021.2005/JCM.43.8.4015.4021.2005 16081944 10.1128/JCM.43.8.4015-4021.2005 PMC1233981

[28] Fang RD, Li X, Hu L, You QM, Li J, et al (2009) Cross-priming amplification for rapid detection of Mycobacterium tuberculosis in sputum specimens. J Clin Microbiol 47: 845–847 DOI10.1128JCM.0152808/JCM.01528-08 19116359 10.1128/JCM.01528-08 PMC2650920

[29] Goldmeyer J, Li H, McCormac M, Cook S, Stratton C, et al (2008) Identification of Staphylococcus aureus and determination of methicillin resistance directly from positive blood cultures by isothermal amplification and a disposable detection device. J Clin Microbiol 46: 1534–1536 DOI10.1128JCM.0223407/JCM.02234-07 18234878 10.1128/JCM.02234-07 PMC2292917

[30] Mugasa CM, Laurent T, Schoone GJ, Kager PA, Lubega GW, et al (2009) Nucleic acid sequence-based amplification with oligochromatography for detection of Trypanosoma brucei in clinical samples. J Clin Microbiol 47: 630–635 DOI10.1128JCM.0143008/JCM.01430-08 19116352 10.1128/JCM.01430-08 PMC2650916

[31] Piepenburg O, Williams CH, Stemple DL, Armes NA (2006) DNA detection using recombination proteins. Plos Biology 4: 1115–1121 DOI10.1371journal.pbio.0040204/journal.pbio.0040204 10.1371/journal.pbio.0040204 PMC1475771 16756388

[32] Puthawibool T, Senapin S, Kiatpathomchai W, Flegel TW (2009) Detection of shrimp infectious myonecrosis virus by reverse transcription loop-mediated isothermal amplification combined with a lateral flow dipstick. J Virol Methods 156: 27–31 DOI10.1016j.jviromet.2008.10.018/j.jviromet.2008.10.018 19022295 10.1016/j.jviromet.2008.10.018

[33] Lee HH, Dineva MA, Chua YL, Ritchie AV, Ushiro-Lumb I, et al (2010) Simple amplification-based assay: A nucleic acid based point-of-care platform for HIV-1 testing. J Infect Dis 201: S65–S72 DOI10.1086650385/650385 20225949 10.1086/650385

[34] Zhou P, Young L, Chen ZY (2010) Weak solvent based chip lamination and characterization of on-chip valve and pump. Biomedical Microdevices 12: 821–832 DOI10.1007s105440109436z/s10544-010-9436-z 20526680 10.1007/s10544-010-9436-z

[35] Rand KH, Rampersaud H, Houck HJ (2011) Comparison of two multiplex methods for detection of respiratory viruses: FilmArray RP and xTAG RVP. J Clin Microbiol 49: 2449–2453 DOI10.1128JCM.0258210/JCM.02582-10 21508156 10.1128/JCM.02582-10 PMC3147849

[36] Böhm S, Olthuis W, Bergveld P (1999) An integrated micromachined electrochemical pump and dosing system. Biomedical Microdevices 1: 121–130 DOI10.1023A1009996407848/A:1009996407848 16281112 10.1023/A:1009996407848

[37] Liu RH, Yang JN, Lenigk R, Bonanno J, Grodzinski P (2004) Self-contained, fully integrated biochip for sample preparation, polymerase chain reaction amplification, and DNA microarray detection. Anal Chem 76: 1824–1831 DOI10.1021ac0353029/ac0353029 15053639 10.1021/ac0353029

[38] Liu RH, Nguyen T, Schwarzkopf K, Fuji HS, Petrova A, et al (2006) Fully integrated miniature device for automated gene expression DNA microarray processing. Anal Chem 78: 1980–1986 DOI10.1021ac0518553/ac0518553 16536436 10.1021/ac0518553

[39] Liu RH, Lodes MJ, Nguyen T, Siuda T, Slota M, et al (2006) Validation of a fully integrated microfluidic array device for influenza A subtype identification and sequencing. Anal Chem 78: 4184–4193 DOI10.1021ac060450v/ac060450v 16771549 10.1021/ac060450v

[40] Lu HW, Roskos K, Hickerson AI, Carey T, Niemz A (2013) System for portable nucleic acid testing in low resource settings. Proc SPIE Microfluidics, BioMEMS, and Medical Microsystems XI 86150 DOI10.111712.2005164/12.2005164

[41] Iwamoto T, Sonobe T, Hayashi K (2003) Loop-mediated isothermal amplification for direct detection of Mycobacterium tuberculosis complex, M-avium, and M-intracellulare in sputum samples. J Clin Microbiol 41: 2616–2622 DOI10.1128JCM.41.6.26162622.2003/JCM.41.6.2616-2622.2003 12791888 10.1128/JCM.41.6.2616-2622.2003 PMC156570

[42] Tan E, Erwin B, Dames S, Voelkerding K, Niemz A (2007) Isothermal DNA amplification with gold nanosphere-based visual colorimetric readout for herpes simplex 2 virus detection. Clin Chem 53: 2017–2020 DOI10.1373clinchem.2007.091116/clinchem.2007.091116 18030699 10.1373/clinchem.2007.091116

[43] Van Ness J, Van Ness LK, Galas DJ (2003) Isothermal reactions for the amplification of oligonucleotides. Proc Natl Acad Sci U S A 100: 4504–4509 DOI10.1073pnas.0730811100/pnas.0730811100 12679520 10.1073/pnas.0730811100 PMC404692

[44] Tan E, Wong J, Nguyen D, Zhang Y, Erwin B, et al (2005) Isothermal DNA amplification coupled with DNA nanosphere-based colorimetric detection. Anal Chem 77: 7984–7992 DOI10.1021ac051364i/ac051364i 16351146 10.1021/ac051364i

[45] Vandeventer PE, Weigel KM, Salazar J, Erwin B, Irvine B, et al (2011) Mechanical disruption of lysis-resistant bacterial cells by use of a miniature, low-power, disposable device. J Clin Microbiol 49: 2533–2539 DOI10.1128JCM.0217110/JCM.02171-10 21543569 10.1128/JCM.02171-10 PMC3147885

