# Supplementary material for: Simple System for Isothermal DNA Amplification Coupled to Lateral Flow Detection
Source: PLoS One. 2013 Jul 26;8(7):e69355. doi: 10.1371/journal.pone.0069355 (PMC3724848; doi:10.1371/journal.pone.0069355)
Supplement: Table S1 — Oligonucleotide Sequences for LAMP Amplification and NALF Detection. (DOCX) [file pone.0069355.s001.docx]

Table S1: Oligonucleotide Sequences for LAMP Amplification and NALF Detection

| Name | Sequences |
| --- | --- |
| Forward Outer Primer  Backward Outer Primer  Forward Inner Primer  Backward Inner Primer  Forward Loop Primer  Backward Loop Primer  NALF Conjugate Probe  NALF Test Line  NALF Control Line | GCGATATCTGGTGGTCTGC  CCGTGGTTTCGAAAACAGC  AGACCACTCGTACCCGTCGCCGGTGGTTAACGCGCTAT  DIG-sp9-ATGAGAAGTCGGAACCCCTGGGACCGTTGACCCCGTCTTC  biot-TEG-sp18-TTGATCTCGACTTCGAGCC  CCTCAAGCAAGGGGCG  NH2-sp12-d(T)90  anti-Digoxin  biot-TEG-d(A)30 |

DIG = digoxigenin; sp9 = C9 spacer; biot-TEG = biotin-tetra-ethylene glycol; sp18 = C18 spacer; sp12 = C12 spacer
