# Supplementary material for: Simple System for Isothermal DNA Amplification Coupled to Lateral Flow Detection
Source: PLoS One. 2013 Jul 26;8(7):e69355. doi: 10.1371/journal.pone.0069355 (PMC3724848; doi:10.1371/journal.pone.0069355)
Supplement: Table S2 — Oligonucleotide Sequences for EXPAR Amplification and NALF Detection. (DOCX) [file pone.0069355.s002.docx]

**Table S2: Oligonucleotide Sequences for EXPAR Amplification and NALF Detection**

| Name | Sequences |
| --- | --- |
| Fingerprinting site  First stage template  Second stage template  NALF conjugate probe  NALF test line probe  NALF control line probe | 5’- GAGTCCAGTATTTGGTCGTCTGTCCTGCGTAGCGACTC -3’  5’- ATTTGGTCGTCGCA*GACTC*ATTT**G**G**T**CGT -3’  5’- ACCGGGCAGATTCGGCCCACTTCCCGCA*GACTC*ATTTGGTCGT -3’  5’- [AmC6]TTTTTTTTTACCGGGCAGATT -3’  5’- CGGCCCACTTCCTTTTTTTTT-sp18-biotin -3’  5’- biotin-sp18-AATCTGCCCGGTAAAA -3’ |

Finger printing site located at position 2574824 within the *M.tb* H37Rv reference genome (accession # CP003248.1). Template design: Sequences underlined with a solid line depict the trigger complement sequence X’, italic letters represent the nicking enzyme recognition site, and bold letters mark the location of locked nucleic acid. Dotted lines indicate the complementary reporter sequence (Y’) used for lateral flow detection. AmC6 = 5’ amine-modified with C6 spacer; sp18 = C18 spacer
