# Supplementary material for: Simple System for Isothermal DNA Amplification Coupled to Lateral Flow Detection
Source: PLoS One. 2013 Jul 26;8(7):e69355. doi: 10.1371/journal.pone.0069355 (PMC3724848; doi:10.1371/journal.pone.0069355)
Supplement: Text S1 — General Reaction Conditions for Isothermal Amplification Coupled to NALF Detection in the Cartridge. (DOCX) [file pone.0069355.s003.docx]

General Reaction Conditions for Isothermal Amplification Coupled to NALF Detection in the Cartridge:

Reaction conditions for isothermal LAMP targeting the M.tb gyrB genomic region were modified from Iwamoto et al (2003). The 100 µL LAMP master-mix contained 20 mM Tris-HCl, pH 7.9, 15 mM ammonium sulfate, 30 mM potassium chloride, 0.005% Triton X-100, 2 mM magnesium chloride, 2 mM magnesium sulfate, 400 µM each dNTP, 1 M betaine, 200 µg/mL bovine serum albumin, 2.4 µM Forward Inner Primer (FIP), 1.6 µM Backward Inner Primer (BIP, DIG-labeled), 0.2 µM of each outer primer (F3 and B3), 0.4 µM biotinylated Forward Loop Primer (bio-LF), 0.8 µM Backward Loop Primer (LB),10 ng of purified human genomic DNA and 20 U of Bst DNA Polymerase large fragment.

EXPAR was carried out in a 75 µl reaction mixture containing 0.3 units/µL Nt Bst nicking enzyme, 0.03 units/µL Bst polymerase, 0.24 mM dNTPs, 1.6 mg/mL BSA, 2 mM magnesium chloride, 1x SYBR Green I, 20mM Tris pH 7.9, 15 mM ammonium sulfate, 30 mM potassium chloride, 0.005% Triton X-100, 10 ng human genomic DNA, 25 nM first stage EXPAR template, and 50 nM second stage EXPAR template.

References:

Iwamoto T, Sonobe T, Hayashi K (2003) Loop-mediated isothermal amplification for direct detection of Mycobacterium tuberculosis complex, M-avium, and M-intracellulare in sputum samples. J. Clin. Microbiol. 41, 2616-2622.
